# Prevalence and contribution of respiratory viruses in the community to rates of emergency department visits and hospitalizations with respiratory tract infections, chronic obstructive pulmonary disease and asthma

**DOI:** 10.1371/journal.pone.0228544

**Published:** 2020-02-06

**Authors:** Imran Satia, Ruth Cusack, Justina M. Greene, Paul M. O’Byrne, Kieran J. Killian, Neil Johnston

**Affiliations:** 1 Firestone Institute for Respiratory Health, St Joseph’s Healthcare, Hamilton, Canada; 2 University of Manchester, Division of Infection, Immunity and Respiratory Medicine, Manchester Academic Health Science Centre, Manchester, United Kingdom; 3 McMaster University Department of Medicine, Hamilton, Canada; National Yang-Ming University, TAIWAN

## Abstract

**Background:**

The individual and combined contribution of viral prevalence in the community to Emergency Department (ED) visits and hospitalizations with respiratory tract infections (RTIs), chronic obstructive pulmonary disease (COPD) and asthma is unclear.

**Methods:**

A retrospective analysis on daily viral positive tests and daily ED visits and hospitalizations between 01/01/2003 to 31/12/2013 in Ontario, Canada. Viral data was collected from the Centre for Immunization and Respiratory Infectious Diseases (CIRID). The Canadian Institute for Health Information reports daily ED visits and hospitalizations for RTIs, COPD and asthma as a primary diagnosis.

**Results:**

There were 4,365,578 ED visits with RTIs of which 321,719 (7.4%) were admitted to hospital; 817,141 ED visits for COPD of which 260,665 (31.9%) were admitted and 649,666 ED visits with asthma of which 68,626 (10.6%) were admitted. The percentage of positive tests to influenza A and B, respiratory syncytial virus (RSV), parainfluenza and adenovirus prevalence explained 57.4% of ED visits and 63.8% of hospitalizations for RTI, 41.4% of ED visits and 39.2% of hospitalizations with COPD but only 1.5% of ED visits and 2.7% of hospitalizations for asthma. The further addition of human metapneumovirus, rhinovirus and coronavirus over the final 3 years accounted for 66.7% of ED visits and 74.4% of hospitalizations for RTI, 52.5% of visits and 48.2% of hospitalizations for COPD, and only 13.3% of visits and 10.4% of hospitalizations for asthma.

**Conclusions:**

Community respiratory viral epidemics are major drivers of ED visits and hospitalizations with RTIs and COPD but only a modest contributor to asthma.

## Introduction

Viruses cause respiratory tract infections (RTIs), which if severe, can lead to a visit to the Emergency Department (ED) and hospitalization. This places a large burden on healthcare services in primary and secondary care but also puts patients with chronic lung diseases such as chronic obstructive pulmonary disease (COPD)[1] and asthma at risk of an exacerbation[2, 3]. During epidemics, viruses can also lead to death as has been demonstrated with influenza, respiratory syncytial virus (RSV) [4, 5], particularly in the young and elderly[6–8]. Therefore, identifying viruses and monitoring the severity of their effects will always remain a major scientific and clinical endeavor.

The Canadian Respiratory Virus Detection Surveillance program consists of a network of laboratories, hospitals, physician offices and provincial and territorial ministries of health. The aim of the program is to detect and respond to outbreaks, use data on viruses to improve and inform health programs and policies, and to ensure information gathered in Canada supports international monitoring and is ready in case of any global threats and outbreaks. The surveillance system was setup in 2003 using multiplex polymerase chain reaction (PCR) to detect 5 viruses: Influenza A and B, RSV, parainfluenza and adenovirus and 3 viruses were added in 2010: human metapneumovirus (hMPV), human rhinovirus (hRV) and coronavirus. Data is available to the public on a weekly basis up until the present time.

The Canadian surveillance program thus provides a unique opportunity to investigate the prevalence of multiple viruses in a real-world setting where the test is routinely performed as part of free universal healthcare. Furthermore, the Canadian Institute of Health Information also report the daily numbers of visits to the emergency department (ED) and number hospitalized for asthma, chronic obstructive pulmonary disease (COPD) and respiratory tract infections (RTI).

Therefore, the objective of this study was to retrospectively investigate the prevalence and contribution of respiratory viruses on daily ED visits and hospitalizations with RTIs, COPD and asthma in an unselected population in Ontario, Canada over a period of 11 years. The yearly and seasonal trend was also explored over the study period.

## Materials and methods

### Data acquisition

“The Respiratory Virus Detection Surveillance System” collects and reports to the Centre for Immunization and Respiratory Infectious Diseases (CIRID) the number of daily tests performed and the number of tests positive for respiratory viruses using multi-plex PCR from laboratories across Canada. The data is reported on a weekly basis for respiratory viruses and were obtained for the province of Ontario. Data were available for Influenza A and B, RSV, parainfluenza and adenovirus for the full study period, hMPV, hRV and coronavirus were added to the testing program in 2010.

Daily ED visits and hospitalizations for asthma (ICD-10 J45), COPD (J40-44, J47) and RTI (J00-J06, J09-J18, J20-22) as a primary diagnosis were obtained from the Canadian Institute for Health Information for the calendar years 2003 to 2013 by date (11 years). Population data were obtained from Statistics Canada. Data for asthma prevalence in Ontario were obtained from Statistics Canada and The Ontario Asthma Surveillance Information System (OASIS).

### Virus detection

Nasopharyngeal swabs were performed on individuals presenting in the community to a health care practitioner in Ontario. Multiplex PCR was performed for the viruses described above. The number of positive tests to each virus was expressed as a percentage of the total number of tests performed and the value obtained for each week was assumed to apply for each day of the previous week.

### Statistical analysis

The population of Ontario increased from 12,251,405 in 2003 to 13,606,541 in 2013. The daily ED visits and hospitalizations were expressed per 10^5^ of the population. The daily rates for ED visits and hospitalizations with RTI, COPD and Asthma were transformed using the natural log (e) of the rates to achieve normal distribution and subsequently used in all analyses. Influenza A & B, RSV, parainfluenza, adenovirus was studied over 11 years (Period A) and Influenza A & B, RSV, parainfluenza, adenovirus, hMPV, hRV and coronavirus over the final 3 years (Period B). The contribution of individual viruses was performed using the Pearson correlation co-efficient. The collective contribution of influenza A & B, RSV, parainfluenza, adenovirus, human metapneumovirus (hMPV), human rhinovirus (hRV) and coronavirus was addressed using multiple regression analysis and the relative strength of each virus to the total variability as the standardized beta value. All analyses were performed in TIBCO Statistica (Academic Package v13.2).

## Results

### Daily ED visits and hospitalizations

**Fig 1:**
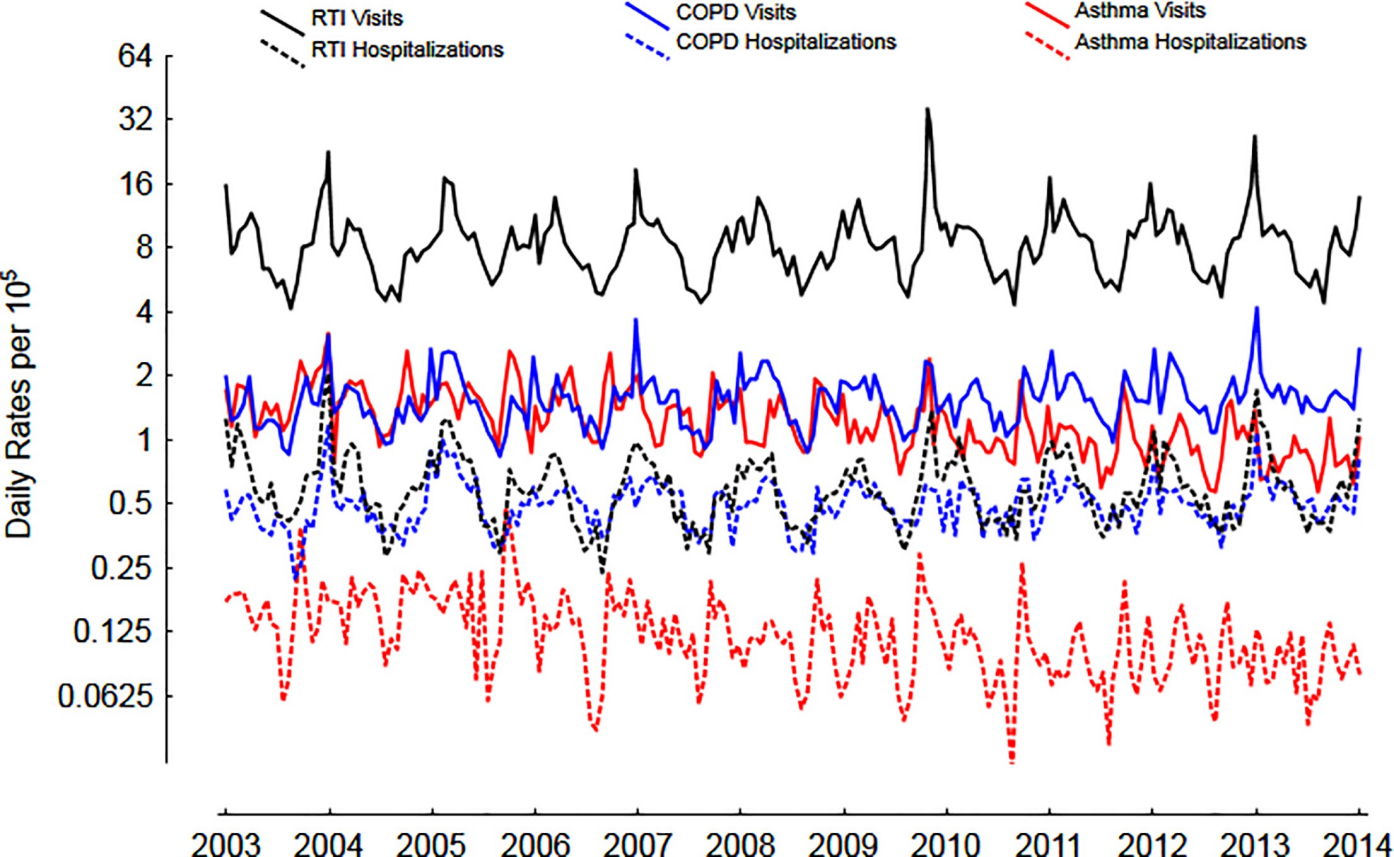
Daily rates per 10^5^ of ED visits and hospitalizations for RTIs, asthma and COPD over 11 years. Y-axis expressed on a log_2_ scale.

**Fig 2:**
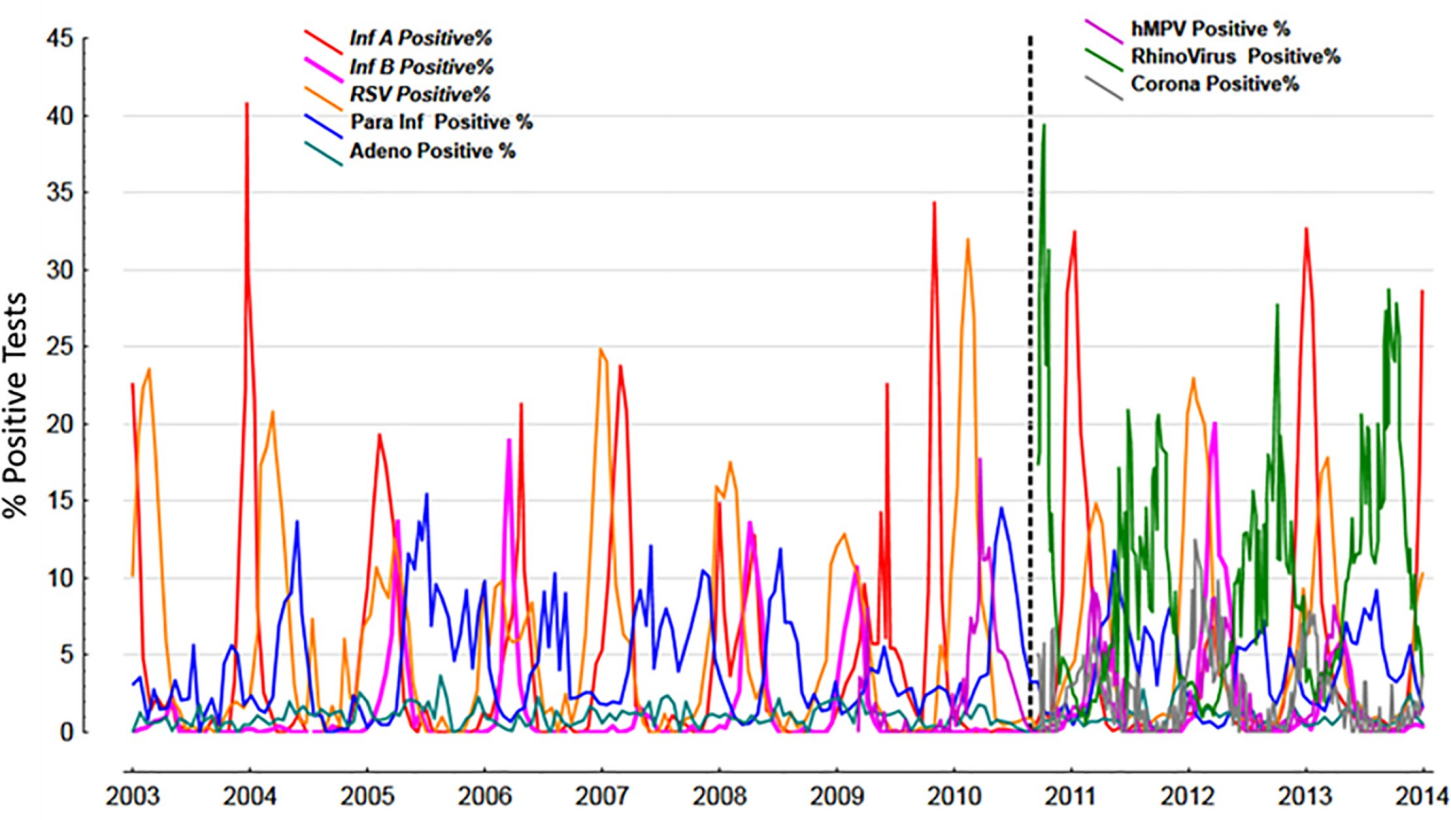
The % positive tests of viruses over the 11 years period. The dashed line represents the period after which an additional 3 viruses were added to the multiplex PCR panel.

The mean number of ED visits and hospitalizations was highest for RTIs, followed by COPD and asthma, however, the % of hospitalized was highest for COPD, followed by asthma and RTIs (Table 1). The daily trends over the entire study period is shown in Fig 1. The prevalence of % positive tests to viruses in the community are shown in Fig 2.

**Table 1:** Numbers of ED visits, hospital hospitalizations and percentages of ED visits hospitalized for respiratory tract infections, asthma and COPD by day of the study between 2003 to 2013 (n = 4018).

Variable	Study Total	Mean Daily Number (S.D.)	Daily Minimum	Daily Maximum
**Emergency Department Visits**				
RTI	4365578	1087 (426.8)	467	6287
COPD	817141	203.4 (61.9)	84	649
Asthma	649666	161.7 (52.9)	63	503
**Hospitalizations**				
RTI	321719	80.1 (31.5)	29	267
COPD	260665	64.9 (17.3)	21	186
Asthma	68626	17.1 (8.3)	1	101
**% of Hospitalizations per ED Visit**				
RTI	30011	7.5 (1.6)	3.5	15.0
COPD	130993	32.6 (5.5)	15.1	61.9
Asthma	41943	10.4 (3.2)	1.3	22.9

### Contribution of individual viruses to ED visits and hospitalizations

The daily % positive response of each virus was correlated with ED visits and hospitalizations for 5 viruses over 11 years (Table 2) and 8 viruses over 3 years (Table 3). ED visits and hospitalizations for RTIs and COPD were strongly and similarly influenced by influenza A, followed by RSV, coronavirus, hMPV and then influenza B. ED visits and hospitalizations for asthma were not influenced by any of the viruses with a minor contribution from hRV (r = 0.21, and r = 0.14 respectively).

**Table 2:** Contribution of individual viruses to the log_e_ daily rates per 10^5^ of the population over 11 years. Univariate correlation matrix showing Pearson r.

Daily Events	Inf A % positive	Inf B % positive	RSV % positive	Para Inf % positive	Adeno % positive
**Emergency Department Visits**					
**RTI**	0.61	0.33	0.49	-0.29	-0.10
**COPD**	0.51	0.35	0.35	-0.27	-0.14
**Asthma**	0.06	-0.01	0.01	-0.10	-0.09
**Hospitalizations**					
**RTI**	0.58	0.26	0.63	-0.35	-0.14
**COPD**	0.50	0.33	0.37	-0.18	-0.10
**Asthma**	0.07	0.02	0.06	-0.15	-0.11

**Table 3:** Contribution of individual viruses to the log_e_ daily rates per 10^5^ of the population over 3 years. Univariate correlation matrix showing Pearson r.

Daily Events	Inf A % positive	Inf B % positive	RSV % positive	Para Inf % positive	Adeno % positive	hMPV % positive	hRV % positive	Corona % positive
**Emergency Department Visits**								
**RTI**	0.65	0.31	0.61	-0.41	0.10	0.35	-0.46	0.53
**COPD**	0.57	0.27	0.42	-0.37	-0.07	0.31	-0.20	0.41
**Asthma**	0.08	0.03	-0.01	-0.18	-0.08	0.03	0.21	0.03
**Hospitalizations**								
**RTI**	0.57	0.27	0.42	-0.37	-0.07	0.31	-0.20	0.41
**COPD**	0.56	0.23	0.43	-0.28	-0.07	0.35	-0.27	0.40
**Asthma**	0.05	0.06	0.05	-0.20	-0.04	0.07	0.14	0.06

### Contribution of all viruses combined to ED Visits and Hospitalization with RTIs

**Fig 3:**
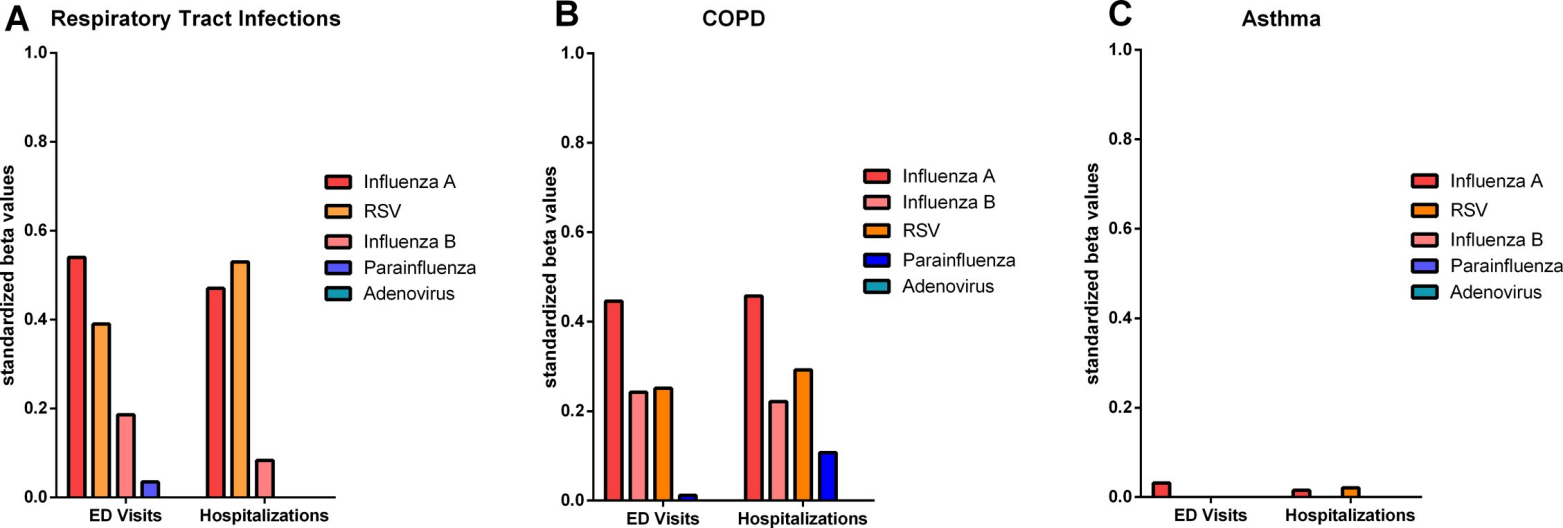
**Contribution of individual viruses to the variability in ED visits and hospitalizations with RTIs (A), COPD (B) and Asthma (C) for 5 viruses over an 11-year period (Period A).** Values on y-axis represent the standardized beta values. Where there was no contribution of virus at reducing the variability in a multivariate model, a value of 0 was assigned.

**Fig 4:**
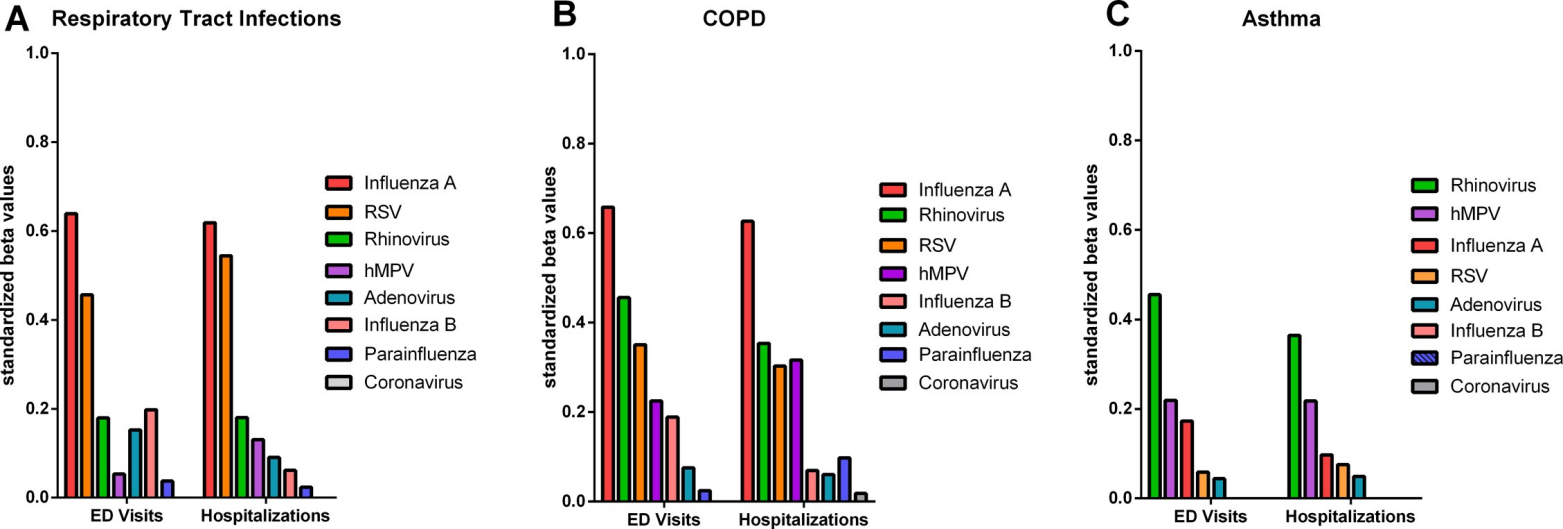
**Contribution of individual viruses to variability in ED visits and hospitalizations with RTIs (A), COPD (B) and Asthma (C) for 8 viruses over a 3-year period (Period B).** Values on y-axis represent the standardized beta values. Where there was no contribution of virus at reducing the variability in a multivariate model, a value of 0 was assigned.

The combination of % positive tests to influenza A & B, RSV, parainfluenza and adenovirus to the daily rates of ED visits and hospitalizations with RTI explained 57.4% of the daily variance in ED visits with RTI and explained 63.8% of the daily variance in RTI hospitalizations in period A. Influenza A & B, RSV, parainfluenza, adenovirus, hMPV, hRV and coronavirus explained 66.7% of the daily variance in ED visits with RTI and 74.4% of the daily variance in hospital hospitalizations over the final 3 years, period B. The relative influence of individual viruses contributing to the variability is shown in Figs 3A and 4A for the 5 viruses over the 11-year period and the 8 viruses over 3 years, respectively. Most of the variance in RTIs was explained by Influenza A, RSV, and hRV.

### Contribution of all viruses combined to ED Visits and Hospitalization with COPD

The combination of % positive tests to Influenza A & B, RSV, paraInfluenza and adenovirus to the daily rates ED visits and hospitalizations with COPD explained 41.4% of the daily variance in ED visits with COPD and explained 39.2% of the daily variance in COPD hospitalizations over the total 11 years (period A). Influenza A & B, RSV, parainfluenza, adenovirus, hMPV, hRV and coronavirus explained 52.5% of the daily variance in ED visits with COPD and 48.2% of the daily variance in hospitalizations over the final 3 years (period B). The relative influence of individual viruses contributing to the variability is shown in Figs 3B and 4B for the 5 viruses over the 11-year period (period A) and the 8 viruses over 3 years (period B). Similar to RTIs, most of the variance explained was due to influenza A, RSV, and hRV.

### Contribution of all viruses combined to ED visits and hospitalization with asthma

The combination of % positive tests to influenza A & B, RSV, paraInfluenza and adenovirus to the daily rates ED visits and hospitalizations with asthma explained 1.5% of the daily variance in ED visits with Asthma and explained 2.7% of the daily variance in asthma hospitalizations over the 11 years. Influenza A & B, RSV, parainfluenza, adenovirus, hMPV, hRV and coronavirus explained 13.3% of the daily variance in ED visits with asthma and 10.4% of the daily variance in hospital hospitalizations over the final 3 years. The relative influence of individual viruses contributing to the variability is shown in Figs 3C and 4C for the 5 viruses over the 11-year period and the 8 viruses over3 years. Most of the variance remained unexplained by the prevalence of viruses in the community, except for a modest contribution from hRV and hMPV.

### Yearly and seasonal variations in ED visits and hospitalizations for RTIs, asthma and COPD

**Fig 5:**
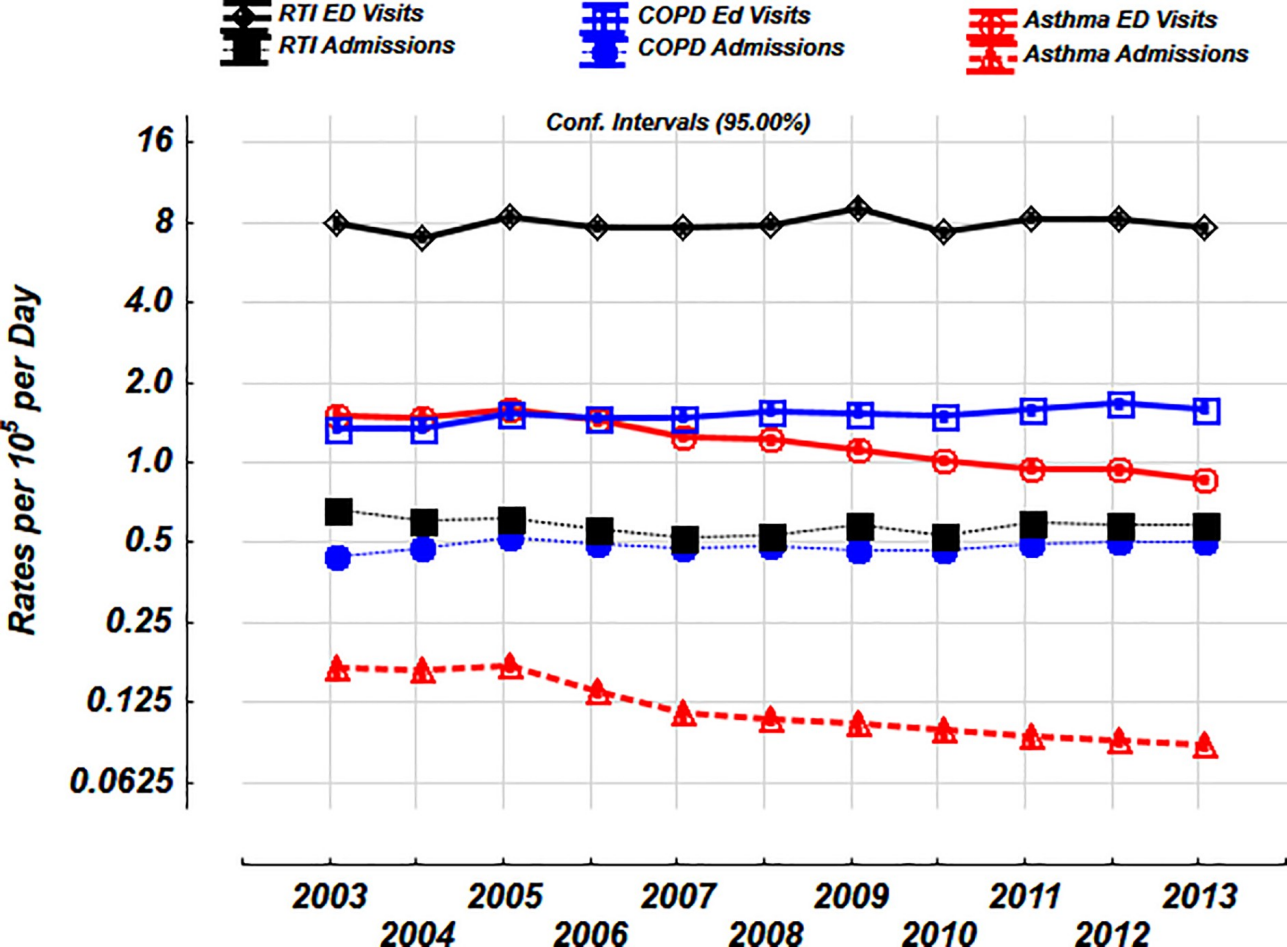
Yearly trends in ED visits and hospitalizations with RTIs, COPD and asthma over 11 years (Period A). Values shown are mean and 95% confidence intervals. Daily rates per 10^5^ expressed on a log_2_ scale.

The daily rates of ED visits and hospitalizations with asthma declined by almost 45% between 2003 and 2013 [1.51 per 10^5^ (95% C.I. 1.47–1.55) to 0.86 per 10^5^ (0.84–0.88), 0.17 per 10^5^ (0.16–0.18) to 0.09 per 10^5^ (0.08–0.09), respectively, Fig 5]. There were no substantial yearly trends for the daily rates of ED visits and hospitalizations for RTIs [8.00 per 10^5^ (7.72–8.29) to 7.62 per 10^5^ (7.37–7.87), 0.67 per 10^5^ (0.64–0.70) to 0.58 per 10^5^ (0.56–0.61, respectively, Fig 5]. There was a 19% increase in the daily rates of ED visits and 16% increase in hospitalizations for COPD over 11 years [1.35 per 10^5^ (1.31–1.39) to 1.60 (1.56–1.65), 0.44 per 10^5^ (0.43–0.45) to 0.51 per 10^5^ (0.49–0.52) respectively, Fig 5].

**Fig 6:**
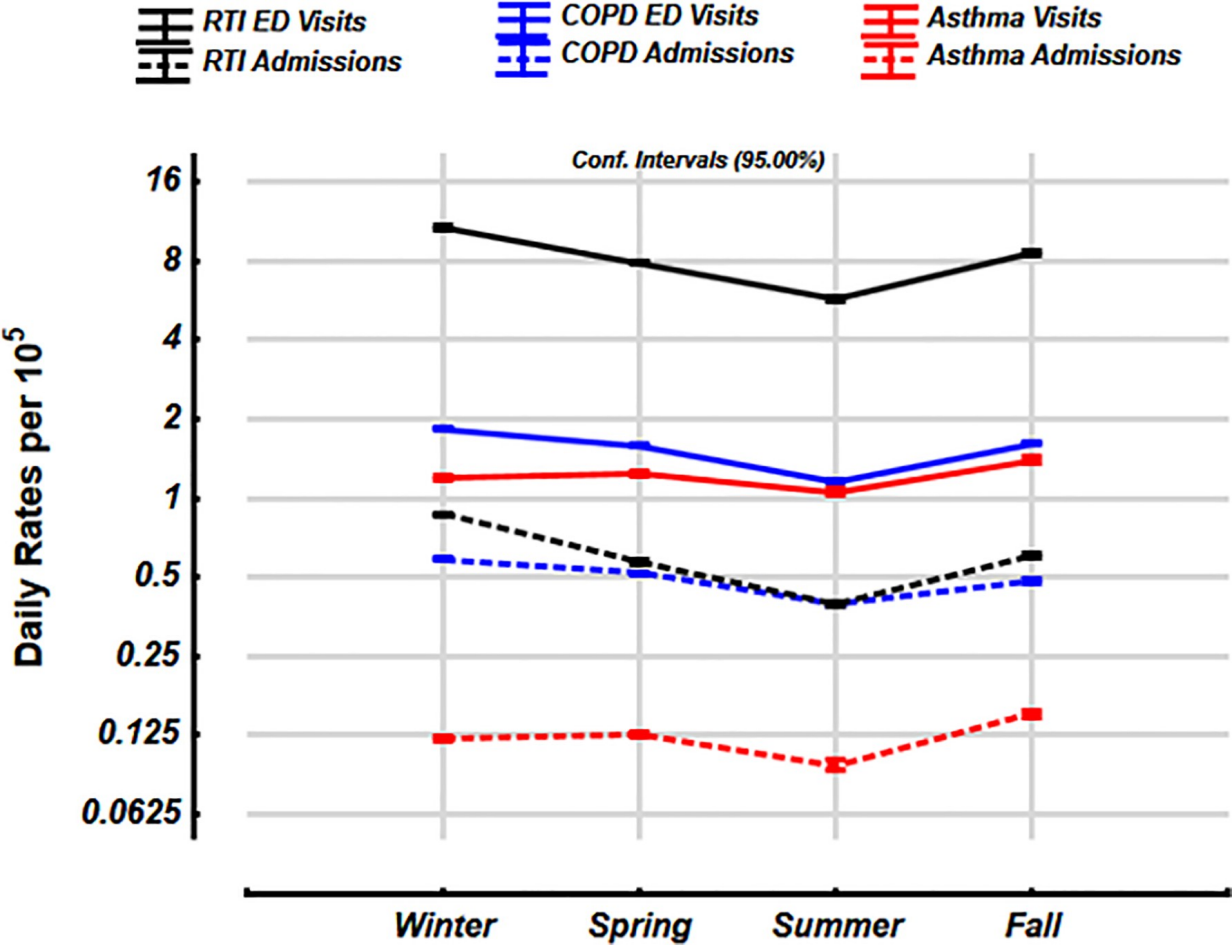
Seasonal trends in ED visits and hospitalizations with RTIs, COPD and asthma over 11 years (Period A). Values shown are mean and 95% confidence intervals. Daily rates per 10^5^ expressed on a log_2_ scale.

The daily rates of ED visits for RTIs and COPD were substantially seasonal with highest rates in the winter [10.7 per 10^5^ (95% C.I. 10.55–10.87) and 1.84 per 10^5^ (1.81–1.87) respectively, Fig 6] and lowest in the summer [5.72 per 10^5^ (5.66–5.79) and 0.59 per 10^5^ (0.58–0.60), respectively]. The rates in the other seasons are also shown in Fig 6. The daily rates for ED visits and hospitalizations for asthma were highest in the fall [1.39 per 10^5^ (1.37–1.72), and 0.15 per 10^5^ (0.146–0.154), respectively] and the lowest in the summer (1.05 per 10^5^ (1.03–1.08), and 0.095 per 10^5^ (0.091–0.096) respectively].

## Discussion

The approach adopted in this study is novel in that we begin with the prevalence of positive tests for a particular respiratory virus (or viruses) in the community on any given day and its contribution to ED visits and hospitalizations with RTI, COPD and asthma over an 11-year period using a multiplex PCR technique. The key findings of this study were: i) ED visits and hospitalizations were highest for RTIs and COPD and follow similar daily patterns, ii) between 41% and 74% of RTIs and COPD related ED visits and hospitalizations can be attributed to respiratory viruses, iii) asthma related visits and hospitalizations were the lowest, with a 45% reduction over the 11 year period and, iv) only human rhinovirus had a minor contribution to asthma.

The case for a causative role for viral respiratory infections in causing exacerbations of COPD and RTIs is strong and explains the concordant temporal relationship with the prevalence of the common respiratory viruses in the community. Our data is in keeping with other studies demonstrating COPD exacerbations coincide with the prevalence of respiratory viruses[9, 10] and subjects who were serially monitored in the community, respiratory viruses are detected in 40% of exacerbations[11, 12] and as high as 60% in a hospital inpatient setting[10, 13]. The higher proportion of patients hospitalized with COPD compared to with RTIs in our study is likely due to a number of disease processes and risk factors such as the presence of chronic airflow limitation, emphysema, smoking, ageing, and associated co-morbidities such as heart failure.

In contrast, viral epidemics and ED visits and hospitalizations with asthma were not synchronous and, except for hRV, did not contribute much to ED visits and hospitalization.

Numerous studies have previously shown 50–85% of asthma exacerbations are reported to coincide with the detection of respiratory viruses[14–16], however they were mainly in cohort studies of children with asthma and often with intensive community monitoring longitudinally[17]. The average detection rates of viruses in incidental studies during exacerbations of asthma were much lower at 24%, with 32% in children and only 13% in adults[18]. Furthermore, virus detection was more prevalent when asthmatics were more symptomatic with wheeze, likely due to a more severe acute illness[18]. Therefore, the low rates of % positive viruses in our study could be explained by the fact that this was an unselected population of the whole of Ontario with children and adults, ED visits likely represent less severe illness in asthma compared with COPD.

Our finding that hRV is the dominant virus in asthma is in keeping with previous data that showed hRV is detected in 33% of adults with exacerbations[19] and two-thirds of children presenting to the ED with an asthma attack were due to hRV[20]. A recent pooled meta-analysis of 60 studies across all ages and continents found the prevalence of respiratory viruses associated with asthma exacerbations was <15%, except hRV which was 42%[21]. Although hRV had the highest prevalence in both children and adults, there were some notable difference: children were more positive for RSV and hRV, whilst adults were more positive for influenza, coronavirus and parainfluenza. These age-related changes in viral detection rates was also evident during the yearly cycle, where children <3 month were mainly positive between the months of Jan-March, whilst those aged between 3–18 were positive in Sept-Nov[22]. Human rhinovirus also occurs in seasonal epidemics in our data, particularly in seasons where influenza and RSV epidemics are much lower. Previously 20% of all hospital admissions of school aged children in Canada were shown to occur in September, attributed predominantly to hRV[23, 24]. Importantly, hRV has recently also been linked with mortality in asthmatics, elderly and in those in long term care facilities [25–27]. It is likely that factors related to hRV species and type [28], along with age, host risk factors determine the severity of symptoms on presentation to the ED may explain the lack of overall relationship to viral epidemics in our data. Data relating to the effects of age and gender in our dataset is not yet to be fully available but will add an important dimension to the current data.

Possible explanations for the low rates of ED visits and hospitalizations for asthma and the 45% fall over 11 years is that i) treatment for asthma with inhaled corticosteroids with or without long acting β2 agonists has improved or ii) the prevalence of asthma has decreased or iii) active cases have decreased across Canada. The Ontario Asthma Surveillance Information System (OASIS) estimated the actual prevalence of asthma at 12.6% in 2003 and 15.3% in 2013, an increase of 22%[29]. This is calculated using a health administrative data case definition of ≥ 2 outpatient claims in 2 consecutive years or ≥ 1 hospitalization(s) for asthma. The Public Health Agency of Canada (PHAC) estimates that the age-standardized prevalence rates of ‘active’ cases, defined as at least one physician claim or ≥ 1 hospitalizations(s) among Canadian in 2003 was 3.3% and this decreased to 2.8%, representing a fall in incidence rates from 723 per 10^5^ to 499 per 10^5^ [30] This suggests that although prevalence of asthma is increasing, the healthcare utilization is decreasing, likely due to improved care and treatment.

There are some important limitations worth noting. First, this analysis was done on 2 separate databases which are unlinked and hence a positive virus detection is not linked with the individual attending the ED. However, the accuracy and validity of the database can be corroborated by the peak in ED visits and hospitalizations with RTIs but not COPD seen in 2009 due to the H1N1 epidemic. Second, the database is based on coding performed by physicians and administrators in the healthcare system, hence there is a potential for miss-classification. Third, the study was not designed to investigate co-infection with multiple viruses. Fourth, as this is a population-based database, we do not have access to additional potentially confounding variables such as medical co-morbidities, allergy status, and medication usage, however, we are currently in the process of acquiring the data for age and gender. Fifth, we do not have information about the day the clinical sample for virus testing was taken in relation to the start of the symptoms. It is possible that virus detection rates may be able altered depending on the day the test was done. Sixth, we do have quantitative PCR data for viral loads, which may be useful at investigating the strength of relationship between virus, severity of illness and hospitalizations. Seventh, each hospital performs nasopharyngeal swabs on the individual patients at their respective sites and different multiplex PCR panels and assays are used for virus testing as universal use of a single multiplex panel is unlikely to be achieved. Each laboratory is licensed in Ontario and are required to undergo quality assurance, auditing and proficiency testing conducted by The Institute for Quality Management in Healthcare.

## Conclusions

Influenza A, RSV and hRV viruses can explain the majority of variability in ED visits and hospitalizations due to RTIs and COPD. Human RV, hMPV and influenza only has a modest contribution to asthma visits and hospitalizations.

## Abbreviations

CIRD: Centre for Immunization and Respiratory Infectious Diseases
COPD: chronic obstructive pulmonary disease
ED: Emergency Department
hMPV: human metapneumovirus
hRV: human rhinovirus
ICD: international classification of disease
MersCoV: Middle East respiratory syndrome coronavirus
OASIS: Ontario Asthma Surveillance Information System
PCR: polymerase chain reaction
RTIs: respiratory tract infections
